# Use of complementary and alternative medicine by patients with end-stage renal disease on haemodialysis in Trinidad: A descriptive study

**DOI:** 10.1186/s12906-017-1755-7

**Published:** 2017-05-04

**Authors:** Mandreker Bahall

**Affiliations:** 1 Arthur Lok Jack Graduate School of Business, University of the West Indies, St. Augustine, Trinidad and Tobago; 2 School of Medicine, University of the West Indies, St. Augustine, Trinidad and Tobago; 3 House #57 , Calcutta Road Number 3, McBean, Couva, Trinidad, LP 62 Trinidad and Tobago

**Keywords:** Adverse effect, Complementary and alternative medicine, Haemodialysis, Prevalence, Treatment

## Abstract

**Background:**

Despite the paucity of scientific evidence, complementary and alternative medicine (CAM) is widely used for the prevention and treatment of illness, holistic care, and counteracting the adverse effects of conventional medicine (CM). This study investigates the use of CAM by patients with end-stage renal disease (ESRD) on haemodialysis.

**Methods:**

This quantitative study was conducted from November 1, 2014 to December 31, 2014 in the haemodialysis unit at San Fernando General Hospital (San Fernando, Trinidad). Face-to-face questionnaire-based interviews were held with101of 125 eligible patients (response rate, 80.5%) at the chairside during haemodialysis. The completed questionnaires were entered into a secure computer database. Data analysis included descriptive analysis, χ^2^ tests, and binary logistic regression analysis.

**Results:**

A minority of the patients were CAM users (*n* = 19; 18.8%). All 19 CAM users took medicinal herbs, 78.9% (*n* = 15) used spiritual therapy, and 10.5% (*n* = 2) used alternative systems. Medicinal tea (*n* = 15; 78.9%), garlic *(Allium sativum)* (*n* = 17; 73.7%), and ginger (*Zingiber officinale roscoe*) (*n* = 13; 68.4%) were the most commonly used medicinal herbs. Seven (36.8%) patients used Chinese herbal medicines and 3 (15.8%) patients used *Aloe vera*. All CAM users were willing to use CAM without supervision or monitoring by their doctors while receiving CM. The use of CAM could not be predicted by age, sex, ethnicity, education, religion, marital status, or employment. Nearly all (98%) patients were satisfied with CAM. More than one-third (36.8%) of patients did not disclose their use of CAM to their doctors, who were generally indifferent to such therapy.

**Conclusions:**

The use of CAM by patients with ESRD was relatively infrequent. All patients used medicinal herbs, most patients used spiritual therapy, and a minority of patients used alternative systems. Complementary and alternative medicine was primarily used for spiritual reasons and the likelihood of its use was influenced by family, friends, and other patients. Patients continued using CM with one or more CAM therapies without informing their healthcare providers, which is a major health risk.

## Background

Health outcomes (e.g. life expectancy, quality of life, and patient satisfaction) have steadily improved worldwide over recent decades. This improvement is largely attributed to the use of conventional medicine (CM). However, there are many unfulfilled health expectations [1, 2] and issues with regard to maintaining wellness [3] and treating several chronic, irreversible, and/or incurable diseases such as ischaemic heart disease and end-stage renal disease (ESRD) [1, 4, 5]. Many patients supplement their health care with complementary and alternative medicine. People use CAM for many reasons, including to cure illness [6], to counteract the adverse effects of CM [7], and/or to promote wellness and holistic care [8, 9]. Patients may also resort to CAM because CM is beyond their financial means [10]. Studies in Trinidad and India reveal that a patient’s CAM use is largely based on perception rather than on science or logic [11, 12]. Patients’ expectations of CAM are less likely to be influenced by the limitations of CM than by the desire to be treated in a manner that is beyond the perceived scope of CM. The reported global prevalence of CAM use ranges widely from 9.8% to 76.0% [13]. It is estimated that CAM is used by 38% of adults in the United States of America (USA) [14], 51.8% of adults in the United Kingdom (UK) [15], and 68.9% of adults in Australia [16]. The annual expenditure on CAM in the UK is on the order of 1.47–1.6 billion pounds [17] and that adults in the USA pay $34 billion out-of-pocket costs for CAM annually [18]. The prevalence of CAM use in Trinidad and Tobago is unknown, but appears to be high.

Nonconventional (i.e. traditional) medicine has been used in Trinidad for centuries [19] and includes ‘home medication/remedies’ and unconventional medical practices [20]. For decades, CAM [3] was the major (and sometimes only) form of medical treatment available in Trinidad [21]. Patients with kidney failure who are on haemodialysis as renal replacement therapy experience adverse effects physically, psychologically, mentally, and socially. Depression, stress, and other psychological problems are common in this patient group [22, 23]. Many patients are neglected by family members [24] and few of their needs are acknowledged by conventional medical practitioners [25], which leaves many patients to fend for themselves with limited or no resources [9]. The quality of life of these patients may also be compromised by coexisting conditions such as diabetes mellitus, hypertension, ischaemic heart disease, bone loss, depression, and other psychosocial and economic problems [23, 24]. According to Roberts et al. [26], CM has improved the outcomes in renal patients, including quality of life, mortality, morbidity, and life expectancy. However, many patients feel helpless and lack the motivation to enjoy life or to continue living in a meaningful way. According to the Ministry of Health (Port of Spain, Trinidadand Tobago), 699 patients accessed dialysis services at public and private health facilities in Trinidad in 2012 [27]. Many of these patients struggle to maintain an acceptable quality of life and turn to CAM to manage several of their issues. However, the use of CAM may have negative consequences. The toxic effects of certain herbs are compounded in patients with ESRD because of the loss of the excretory function of the kidneys [28].

Complementary and alternative medicine therapies are of questionable safety and efficacy and may be injurious to health [29–33]. In some situations, CAM is used instead of CM. The perceived benefits of CAM such as the ability to cure disease, treat the adverse effects of CM, and improve quality of life and general well-being provide hope for many CAM users was reported in a study of cardiac patients [11]. This perception of CAM benefits may also be true among dialysis patients. However, scientific scrutiny of CAM [34] is needed with regard to the safety, efficacy, and quality of CAM therapies; the accessibility to CAM agents; and the rational use of CAM [35]. These objectives can be strengthened with appropriate public health policies [35, 36] and legislation [37].

No studies have been conducted on the use of CAM in patients with ESRD on haemodialysis in Trinidad. This study explored CAM use in these patients with regard to the type of CAM; the patients’ reasons for using CAM; and the influences, benefits, outcomes, associations, and predictors of CAM use.

## Methods

**Fig. 1:**
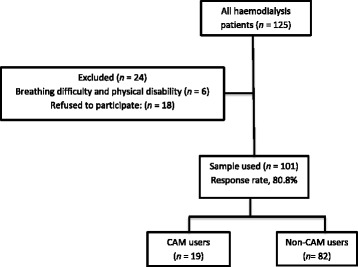
Flow chart of the total patient population, respondents, and CAM users/nonusers. CAM, complementary and alternative medicine

This cross-sectional study was conducted in patients attending for haemodialysis at San Fernando General Hospital (San Fernando, Trinidad) between November1, 2014 and December 31, 2014. The public dialysis unit at this hospital services approximately one-half of the population (600,000 individuals) of Trinidad [38]. Patients who cannot access this free service can have dialysis privately by self-funding or with the assistance of government at private institutions. All consenting patients were recruited for this study (Fig. 1). The eligibility criteria were an age older than 18 years, mental competence (i.e. no cognitive or behavioural problems), ability to communicate verbally, and informed consent. The exclusion criteria included confusion and inability to complete an interview.

The data collection instrument was a questionnaire containing 37 questions (7questions on demographics; 5 questions on ESRD status; and 25 questions on various aspects of CAM use such as type of CAM product, reasons for use, perceived benefits, influences, effects and consequences, source, access to CAM, and satisfaction with CAM). Data on 8 independent variables were collected, including sex, marital status, ethnicity, educational level, employment status, religion, religiosity, and place of residence. The determination of CAM usage was based on patients’ choices from a list of different types of CAM (e.g. herbs, biologically based therapies, alternative systems, spiritual therapy/mind-body systems, physical therapy/body manipulations, energy therapies and other local/indigenous therapies). The types of CAM were based on the definitions used by the National Centre for Complementary and Alternative Medicine (NCCAM), located in Maryland, United States, and practices in Trinidad. Patients who routinely used spices and certain herbs or who took vitamins as a prescribed CM treatment were not considered as using CAM. However, if the spices and herbs were used outside of a doctor’s prescription and for the purpose of medical treatment, then their use was considered CAM use. The instrument (i.e., the 37-item questionnaire) was not validated, although it was pilot-tested for clarification and was used by a similar population among cardiac patients in Trinidad [11]. Face-to-face interviews were conducted with consenting patients at the chairside during dialysis sessions lasting approximately 3–4 h. The data collected were entered in a secure database accessible only to the researcher, an assistant, and a statistician. The statistical analysis was conducted using SPSS, version 20 software (IBM Corp., Armonk, NY, USA) [39] with descriptive and inferential methods. The descriptive methods included frequency distribution tables and graphs, and the inferential methods included tests or equality of proportions, and χ^2^ tests for associations between the use of CAM and selected sociodemographic and other variables (Fisher’s exact test and McNemar’s test for paired proportions, as applicable). Binary logistic regression was used, if necessary, to identify the predictors of CAM use in the study population, based on significant associations identified from socio demographic variables. All hypotheses were tested at the 5% level of significance. Ethical approval to conduct this study was obtained from the ethics committee at the Southwest Regional Health Authority (San Fernando, Trinidad) on September 28, 2014.

## Results

In total, 101 of 125 patients attending the dialysis unit were included in the study (response rate, 80.8%). Most patients were cooperative and happy to have someone to converse with during their long dialysis session. However, some patients felt tired and wanted to rest while they dialyzed, and therefore preferred not to be interviewed. In addition, some patients had a physical disability that precluded their participation. In total, 24 patients were excluded from the study because of communication difficulties, refusal to participate, or inability to complete the interview. All 101 questionnaires were usable. The reliability of the instrument (i.e. Cronbach’s α) was 0.813. Table 1 shows the demographic profile of the respondents: the sex distribution was even, 82 (81.1%) patients were more than 40 years old, 52 (51.5%) patients were married,62 (62.4%) patients were Indo-Trinidadian, 60 (59.4%) patients had at least a secondary school education; 85 (84.2%) patients were unemployed during the data collection period, and 44 (43.6%) patients reported being Christian. The CAM users were in a minority with only 19 (18.8%) patients answering ‘yes’ to the question ‘Have you used complementary alternative medicine?’. The demographic profiles of users and nonusers are shown in Table 2 and are generally similar.

**Table 1:** Sociodemographic characteristics of the patients

Variable	Number	Percent
Sex		
Male	50	49.5
Female	51	50.5
Age (y)		
< 20	1	1.0
21–30	5	5.0
31–40	12	11.9
41–50	27	26.7
51–60	28	27.7
> 60	28	27.7
Marital status		
Single	18	17.8
Married	52	51.5
Widowed	15	14.9
Divorced	9	8.9
Common law	7	6.9
Ethnicity		
Afro-Trinidadian	30	29.7
Indo-Trinidadian	63	62.4
Other (including mixed)	8	7.9
Highest level of education		
Up to primary	41	40.6
Secondary	49	48.5
Tertiary	11	10.9
Employment status		
Employed	16	15.8
Unemployed	85	84.2
Religion		
Hindu	30	29.7
Islam	8	7.9
Christian	44	43.6
Other	17	16.8
None	2	2.0

**Table 2:** Demographic characteristics of CAM users and nonusers

	CAM use status, *n*(%)		
Variable	No CAM	CAM	*P*-value
Sex			
Male	41 (50.0)	9 (47.4)	0.836
Female	41 (50.0)	10 (52.6)	0.836
Age (y)			
< 20	1 (1.2)	0 (0.0)	0.314
21–30	4 (4.9)	1 (5.3)	0.946
31–40	7 (8.5)	5 (26.3)	0.092
41–50	22 (26.8)	5(26.3)	0.946
51–60	21 (25.6)	7 (36.8)	0.352
> 60	27 (32.9)	1 (5.36)	≤0.001
Marital status			
Single	16 (19.5)	2 (10.5)	0.278
Married	38 (46.3)	14 (73.7)	0.017
Widowed	13 (15.9)	2 (10.5)	0.511
Divorced	9 (11.0)	0 (0.0)	≤ 0.001
Common law	6 (7.3)	1 (5.3)	0.727
Ethnicity			
Afro-Trinidadian	26 (31.7)	4 (21.1)	0.318
Indo-Trinidadian	49 (59.8)	14 (73.7)	0.224
Other	7 (8.5)	1 (5.3)	0.584
Employment status			
Unemployed	70 (85.4)	15 (78.9)	0.572
Employed	12 (14.6)	4 (21.1)	0.527
Monthly income (TT$)			
2501–5000	2 (2.4)	0 (0.0)	0.152
5001–10,000	1 (1.2)	0 (0.0)	0.314
No response	79 (96.3)	19 (100.0)	0.078

*CAM* complementary and alternative medicine, *TT$* Trinidad and Tobago dollars

**Table 3:** Frequency of the use of different types of CAM by kidney patients

		Use pattern, *n* (%)		
CAM		Used in past	Used presently	Will use in the future
Herbs				
	Evening primrose *(Oenothera)* oil	1 (5.3)	0 (0.0)	0 (0.0)
	Flaxseed *(Linum usitatissimum)*	4 (21.1)	6 (31.6)	5 (26.3)
	Ginger *(Zingiber officinale r*oscoe*)*	13 (68.4)	12 (63.2)	13 (68.4)
	Ginseng *(Panax)*	6 (31.6)	7 (36.8)	7 (36.8)
	Medicinal tea	15 (78.9)	14 (73.7)	14 (73.7)
	Turmeric *(Curcuma longa)*	6 (31.6)	4 (21.1)	4 (21.1)
	Ginkgo *(Ginkgo biloba)*	3 (15.8)	3 (15.8)	3 (15.8)
	Garlic *(Allium sativum)*	14 (73.7)	11 (57.9)	10 (52.6)
	*Aloe vera*	3 (15.8)	2 (10.5)	2 (10.5)
Biologically based therapies				
	Potassium	4 (21.1)	11 (57.9)	10 (52.6)
	Calcium	11 (57.9)	12 (63.2)	14 (73.7
	Vitamin B complex	18 (94.7)	0 (0.0)	0 (0.0)
	Vitamin A	6 (31.6)	6 (31.6)	9 (47.4)
	Vitamin D	4 (21.1)	3 (15.8)	6 (31.6)
	Vitamin E	4 (21.1)	3 (15.8)	6 (31.6)
	Zinc	1 (5.3)	0 (0.0)	1 (5.3)
	Omega 3	6 (31.6)	3 (15.8)	5 (26.3)
	Sure Cure products	1 (5.3)	0 (0.0)	0 (0.0)
	Folic acid	8 (42.1)	14 (73.7)	13 (68.4)
	Omega XL	4 (21.1)	0 (0.0)	0 (0.0)
	Special diet/supplements	2 (10.5)	15 (78.9)	15 (78.9)
	COQ 10	3 (15.8)	1 (5.3)	2 (10.5)
Alternative systems				
	Chinese herbal medicine	7 (36.8)	6 (31.6)	6 (31.6)
	Indian/Ayurvedicmedicine	Nil	Nil	Nil
	Acupuncture	Nil	Nil	Nil
	Homeopathy	Nil	Nil	Nil
Other (e.g. spiritual therapy/mind-body systems, physical therapy/body manipulations, energy therapies, other indigenous/local therapies)		Nil	Nil	Nil

*CAM* complementary and alternative medicine; *COQ 10* coenzyme Q 10

**Table 4:** Patients’ reasons for deciding to use CAM

Reason	Number	Percent
Disappointed that conventional treatment was not working	8	42.1
Conventional treatment too toxic or damaging	2	10.5
CAM more in keeping with beliefs and inner self	5	26.3
Finding conventional treatment too mechanistic/technological and lacks human touch	1	5.3
Just trying everything that can help	5	26.3
Conventional treatment too expensive	14	73.3

*CAM* complementary and alternative medicine

**Fig. 2:**
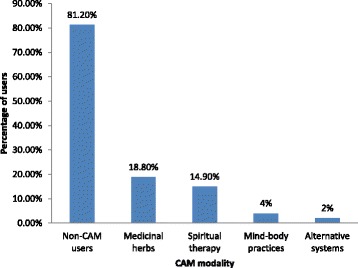
The prevalence of each CAM modality among patients with end-stage renal disease (*n* = 101). CAM, complementary and alternative medicine

Figure 2 shows that all 19 CAM users were taking medicinal herbs, 15 (78.9%) patients also used spiritual therapy and 2 (10.5%) patients used alternative systems. None used energy therapy (e.g. bioelectrics/magnetics, oxygen/ozone treatment). Medicinal tea (*n* = 15; 78.9%), garlic *(Allium sativum)* (*n* = 17; 73.7%), and ginger *(Zingiber officinale roscoe)* (*n* = 13; 68.4%) were the most commonly used medicinal herbs (Table 3). In addition, 7 (36.8%) patients used Chinese herbal medicines and 3 (15.8%) patients used *Aloe vera*.

Faith healing/prayer (*n* = 15; 78.9%) and meditation (*n* = 2; 10.5%) were the only spiritual therapies used by the CAM users. None used visualization/vision therapy, hypnotherapy, psychic therapy, mind-body techniques, divinations/incantations, or folk magic/sorcery (i.e. ‘*obeah’*) to treat their illness. Two patients used alternative systems such as acupuncture, but none used Chinese medicine, Indian/Ayurvedic medicine, or homeopathy. Chiropractic was used by each of 4 (21.1%) patients who used physical therapy/body manipulation to treat their condition. No patient used the following to treat their ESRD: osteopathy/bone manipulation, massage, manual healing (e.g. therapeutic touch), bloodletting, cupping, local surgery/scarification, ritual sacrifice, urine, or folk remedies.

### Benefits and reasons for CAM use

**Fig. 3:**
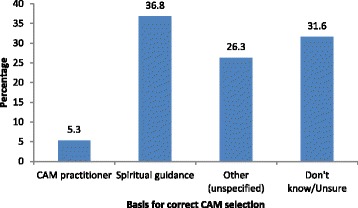
Basis for appropriate CAM use. CAM, complementary and alternative medicine

Fourteen (73.7%) patients claimed to have received some specific benefit from their use of CAM, and some patients provided the reasons for their decision to use it (Table 4). Fourteen (73.3%) patients attributed their decision to use CAM to the expense of conventional treatment. Two patients reported using CAM because conventional treatment was too toxic or damaging, and another patient claimed to have resorted to CAM because conventional treatment was too mechanistic and technological and lacked the human touch. Only one (5.3%) user was dissatisfied with the outcome of CAM. The remaining 16 (84.2%) patients were satisfied, and 2 (10.5%) patients were very satisfied. One user experienced adverse effects (e.g. vomiting and diarrhoea) from CAM use. With regard to how they were able to tell that they were using the right CAM, given the many competing options, only one (5.3%) user said that the decision was based on advice from a CAM practitioner and 6 (31.6%) patients said that they did not know (Fig. 3). None of the patients taking CAM abandoned their CM and all refused to substitute their CM with CAM.

**Fig. 4:**
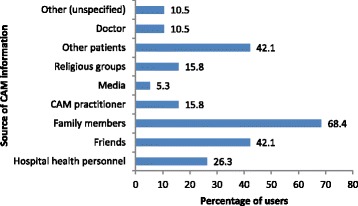
Source of information about CAM. CAM, complementary and alternative medicine

**Fig. 5:**
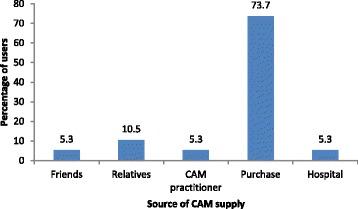
Source of CAM supply. CAM, complementary and alternative medicine

More than one-third (36.8%) of the patients did not inform their CM provider that they were also taking CAM. Three (15.8%) of the 19 CAM users commenced CAM on consultation with an alternative medicine practitioner before going to their medical doctor; however, none informed their medical doctor that they were already using CAM at their first visit. Twelve of the remaining 16 patients who started using CAM when they were already on haemodialysis informed their doctor that they were using it, but 4 patients did not. When the 7 patients who had not informed their CM doctor about their use of CAM were questioned as to why they never did so, 6 patients said that they did not think it was necessary to do so, while one patient reported having made at least one attempt to mention their use of CAM but was ignored by the doctor. Further, use of CAM was medically supervised in only one patient.

### Influences, interactions, benefits, and monitoring

Advice from family was the major factor influencing the decision to use CAM (*n* = 13; 68.4%), and only one (5.3%) patient was introduced to CAM via the media (Fig. 4). The benefits that users hoped to derive from CAM were, as follows (in descending order): improved psychological/emotional well-being (*n* = 10; 52.6%), relief of symptoms/adverse effects associated with CM (*n* = 8; 42.1%), curative treatment of a condition (*n* = 2; 10.5%), and relaxation/sleep (*n* = 1; 5.3%).

Most users purchased their own CAM, while 5.3% obtained it at the hospital (Fig. 5). In response to the statement ‘the more knowledgeable a person is regarding CAM, the more likely CAM will be used by such a person’, 31.6% (*n* = 6) of CAM users agreed, 52.6% (*n* = 10) agreed strongly, and 15.8% (*n* = 3) disagreed. The demographic variables tested were not useful predictors of the likelihood of use of CAM by dialysis patients.

## Discussion

Complementary and alternative medicine is defined as ‘a group of diverse medical and health care systems, practices, and products that are not generally considered part of conventional medicine’ [14]. It includes herbs, dietary supplements, meditation, biofeedback, hypnosis, acupuncture, Ayurveda, homeopathy, naturopathy, Chinese medicine, chiropractic, massage, tai chi, yoga, electromagnetic therapy, kinesiology, reiki, and qigong. In this study, the prevalence of CAM use among haemodialysis patients was relatively low (18.8%). This prevalence was similar to the prevalence (18%) of herbal CAM use among haemodialysis patients in Cincinnati, OH, USA, [40]. The prevalence of CAM use in Palestine was 64.4% in one report [41]. In a tertiary care hospital in India, 26% of patients used CAM, most commonly Ayurveda (30.4%) [42]. The prevalence of CAM use among patients in a chronic renal failure clinic in Turkey was 25.2%, and primarily consisted of body-mind therapies (46.1%) [43]. In Trinidad and the Caribbean, the prevalence of CAM use is low in patients on haemodialysis when compared with its use in other patient subgroups (e.g. cardiac patients (56%) [44] and asthmatic patients (30.4%) [45]). This finding may be partially related to the nature of the renal disease, which can increase the toxicity of some chemicals because of the loss of excretory function of the kidneys. In the present study, most CAM users used medicinal herbs (100%) and spiritual therapy (78.9%). Spiritual therapy is widely practiced, as demonstrated by Finkelstein et al. [46]. We found no association between the use of CAM and age, ethnicity, sex, educational level, or income in patients on haemodialysis. This finding is similar to those obtained by Birdee et al. [8], who found that the use of CAM did not differ significantly, based on sex, race, number of years on dialysis, diagnosis of ESRD, employment status, or educational level of patients with ESRD. Erdoğan et al. [47] also found no relationship with hopelessness.Nearly 20% of all dialysis patients in this study used herbal medications, the most common being herbal tea. This is of concern because many herbs are noted for their toxic effects. The most commonly used herbal CAMs are Chinese herbal tea (31.6%), flaxseed (*Linum usitatissimum*; 31.6%), ginger (*Zingiber officinale roscoe*; 63.2%), ginkgo (*Ginkgo biloba*; 15.8%), ginseng (*Panax*; 36.8%), turmeric (*Curcuma longa*; 21.1%), garlic (*Allium sativum*; 57.9%), and medicinal tea (73.7%) (Table 3). These herbal products may interfere with the CMs used in the treatment of ESRD [48]. This consequence is because ESRD increases the likelihood of herbal, herb-drug, or herb-herb toxicity or interactions or both. Ginkgo causes behavioural changes, bleeding, and ischaemia [49]. Bleeding can be worsened with blood thinners such as aspirin when combined with herbs such as ginkgo [18, 50–52], garlic [52, 53], and flaxseed [54]. However, claims of such adverse interactions with ginkgo and garlic have not been supported in some studies [55, 56]. Turmeric also has blood thinning properties [57]. Based on small nonrandomised trials, several traditional Chinese herbal medicines are associated with significant adverse effects, including nephrotoxicity [58]. Many CAM practices in Trinidad, including vigorous massage therapy, can be detrimental to health [59] and should be avoided in patients with kidney failure. Further, herb-drug interactions can precipitate kidney failure [48]. Some CAM products may have beneficial effects in patients with kidney disease, including prolonging the time to progression and treatment of concomitant complications [60]. Many herbal remedies are also useful for patients experiencing cramps [61] and micro inflammation [62], which are common in patients with ESRD.

In this study, more than 90% of patients were generally satisfied with CAM and claimed that they experienced better health when taking these agents. Only 5.3% reported being dissatisfied. In contrast with the present findings, Barbadoro et al. [63] found that most CAM users reported less satisfaction (60.6%, acupuncture; 69.2%, herbal medicine; 70.8%, homeopathy; 77.8%, manual treatments). In the present study, most (73.7%) CAM users recognised the benefits of CAM, although none stopped using CM. This finding may reflect an appreciation of the value of CM, fear of stopping CM, or lack of complete faith in CAM. The benefits derived from CAM were improvement in psychological/emotional well-being (*n* = 10; 52.6%) and relief of symptoms/side effects associated with CM (*n* = 8; 42.1%). People were discouraged by the mechanistic nature of CM and by its lack of the human touch, financial costs, toxicity, and failure rate (i.e. push factors); they were encouraged by the synchrony between a patient’s beliefs and CAM (i.e. pull factors; Table 4).

The wide variety of uses for CAM identified in other studies was not revealed in this study, which may be because of its relatively small sample size and low proportion of CAM users. The benefits of CAM reported by other investigators are that CAM is ‘natural’ and ‘safe’ [64], is effective [60], has fewer adverse effects [64], and allows a feeling of control, coping, and adjustment [65].

Most patients seemed to have no clear guidelines on the appropriate use of CAM or a rational basis for using it. Only 5.3% reported guidance by a CAM practitioner. Its use continues unregulated in Trinidad and Tobago because of the inadequacy of local regulations [37] and because of the support and encouragement from others, particularly family (68.4%), friends (42.1%), other patients (42.1%), CAM practitioners (15.8%), and doctors (10.5%). Our finding that family members had the most influence on the likelihood of using CAM has been noted by others [64]. Use of CAM is also influenced by social, cultural, economic, and traditional factors [66].

Simultaneous use of CAM and CM comes with a risk of herb-drug interactions. This factor necessitates greater knowledge, understanding, and communication between patients and clinicians (who may be unprepared to manage herb-herb or herb-drug interactions). In this study, at least one-third of patients were either unwilling to disclose their use of CAM to their CM provider or only did so after commencing CAM. Many CM providers were dismissive of CAM and unwilling to entertain a discussion about it. Rao et al. [42] also found that patients were very hesitant to disclose use of CAM to their physician. Furthermore, 72% of patients did not discuss their use of CAM with their doctors and 46% of doctors did not ask patients if they used CAM [64]. High satisfaction levels, high nondisclosure rates, lack of meaningful communication with healthcare providers, and lack of supervision and monitoring of its use makes CAM a major healthcare problem. This lack of communication may not be in the best interest of patients, given that vital information required for effective patient management is lost and given the potential for negative effects. This factor requires attention by policy makers, clinicians, and patients.

The limitations of this study include the selection of patients from a single public dialysis centre. Patients at this clinic tend to be of a lower socioeconomic status and educational level, and therefore may not be fully representative of the population on dialysis in Trinidad. The sample was non-randomised, although every effort was made to choose all consenting patients from this centre to minimise bias. Further, there may be a degree of recall bias. Efforts were made to maintain privacy during interview, although patients may have altered their answers because interviews were conducted at the chair side while they were receiving dialysis where they may have felt uncomfortable about disclosing all information within the hearing of other dialysis patients and staff members.

## Conclusions

The prevalence of CAM use was relatively low in this group of patients with ESRD on haemodialysis. All CAM users took herbs and most users used both herbs and spiritual treatment. There was no association between the use of CAM and age, ethnicity, sex, educational level, or income. The use of CAM was driven by the cost of CM, personal beliefs, willingness to try anything, and perceived benefits such as the human touch associated with CAM. The majority (>90%) of patients were satisfied with CAM and prepared to use it simultaneously with CM. At least one-third of patients in this study used CAM without the knowledge of their doctors or without appropriate medical guidance, and instead received their information primarily from family, friends, and fellow patients. Physicians need to be aware of and ask patients about their use of CAM. This knowledge could prevent potentially dangerous drug-herb or drug-drug interactions caused by the concomitant use of CAM and CM.

## Abbreviations

CAM: Complementary and alternative medicine
CM: Conventional medicine
ESRD: End-stage renal disease
